# Association between human cytomegalovirus infection and incidence of severe orofacial clefts: a series of rare cases

**DOI:** 10.1186/s12903-025-06165-1

**Published:** 2025-05-23

**Authors:** Mohamed Abd-El-Ghafour

**Affiliations:** Department of Orthodontics, Faculty of Dentistry, Cairo University, Cairo, Egypt

**Keywords:** Cleft lip, Facial cleft, Cytomegalovirus, Infants

## Abstract

**Background/Objectives:**

Human cytomegalovirus (HCMV) infection was scarcely reported as a prejudicing factor of CLP incidence. The aim of this study is to report an association between HCMV infection and incidence of severe orofacial clefts.

**Materials/Methods:**

Five infants with severe orofacial clefts were included in the current study. One of the infants is with an oblique facial cleft extended to the left orbit and associated with missing left eyeball. Another infant was with facial cleft extending downward resulting in a clefted lower lip, clefted mandible and clefted tongue. All the patients’ mothers were evaluated for the amount of HCMV IgG in blood samples.

**Results:**

All the five mothers of the included infants had a positive HCMV IgG results in the collected blood samples with the value of 908.8 (± 400.6). The amount of HCMV IgG was higher in cases with more severe clinical presentation.

**Limitations:**

Due to the rarity of cases with severe orofacial clefts, only 5 patients were included. Lake of genetic data and control group are another limitations.

**Conclusion:**

Within the limitation of the rareness of cases with severe orofacial clefts, HCMV infection to mothers before or during pregnancy might be a predisposing factor of incidence of severe orofacial clefts.

## Introduction

Cleft lip and palate is considered as the most common craniofacial anomaly. 1 The severity and extent of the defect varies greatly between patients. 2 It can be very mild, represents as notching in the lip or as a small slit in the uvula and can be severe enough that extends facially producing severe facial disfigurement. 2.

Etiological factors behind the development of orofacial clefts are multiple and not fully proved. As mentioned in the published systematic reviews, 5 possible etiological factors were suggested to be the most related to incidence of oral clefts. Genetic factors^3^, ^4^, maternal negative smoking 5, 6, folic acid deficiency^7^, ^8^, maternal alcohol consumption^9^ and teratogenicity 10, 11, 12 are the most possible reasons of oral cleft occurrence.

HCMV is one of the *Herpesviridae* viruses that causes no symptoms after its infection.^13^ It remains dormant in the infected individuals and stays in the bone marrow derived cells.^13^ HCMV is considered as the most common congenital infections represented as 0.2-2% of live birth.^14^ As mentioned in a systematic review published in 2014,^15^ maternal HCMV seroprevalence was ranged from 84 to 100% and birth prevalence was ranging from 0.6–6.1%.^15^ HCMV can be transmitted from mothers to their infants causing fetal HCMV infection. This could be associated with congenital anomalies affecting the different systemic organs.^16^.

Upon infection with HCMV, especially during pregnancy, several physiological processes can be affected including vascular damage, cell death in developing tissues and neural crest disruption which can secondarily affect the craniofacial development.^16^ The pathogenesis of HCMV infection and its effect in the craniofacial region has been investigated in the literature. Some studies suggested that HCMV hinders the differentiation of the precursor cells 17, 18 while other studies suggested the induction of inflammatory process and prevention of precursor cell proliferation due to its ability of disrupting the cell cycles. 19, 20 Moreover, a study has mentioned that HCMV infection leads to non-tissue fusion, which may be associated with variations in cell signaling pathways accountable for migration, mitosis, apoptosis, and cell differentiation.^21^.

Association between HCMV infection and development of cleft lip and palate was scarcely mentioned in the literature. In the found 3 articles,^14^, ^22^, ^23^ strong association between orofacial cleft occurrence and congenital HCMV infection was reported. None of the published studies discussed the association between severe orofacial cleft development and HCMV infection.

The aim of this article is to present 5 cases with severe orofacial cleft and to report the involvement of HCMV infection of their mothers during pregnancy.

## Materials and methods

Five cases with severe orofacial cleft were presented in the outpatient clinic of the orthodontic department, Cairo University, Egypt. Cases are presented starting from the least to the more severe (Table 1).

**Table 1:** Summary of the amounts of HCMV IgG and clincial features

Case number	Amount of Mothers’ HCMV IgG (UI/ml)	Tessier Classification	Clincal Features	Figure
Case 1	422	0	Median cleft with congenitally missing premaxilla.	1
Case 2	452	0	Median cleft with congenitally missing premaxilla.	2
Case 3	1034	2 & 8	Hemifacial microsomia with skin tags on the left cheek and bilateral complete cleft lip and palate and	3
Case 4	1261	2	Cleft in the lower lip, mandibular osseous cleft and unilateral complete cleft lip and palate in the maxilla.	4
Case 5	1375	3	Oblique facial cleft on the left side including the upper lip and extending upward resulting in complete loss of the left eyeball. Severe deformities are present in the 2 hands in the form of fused and missing fingers. In lower limbs fusion and deformity of the toes of the right leg, in addition to complete absence of the left sole of the foot.	5&6

### Case 1

**Fig. 1:**
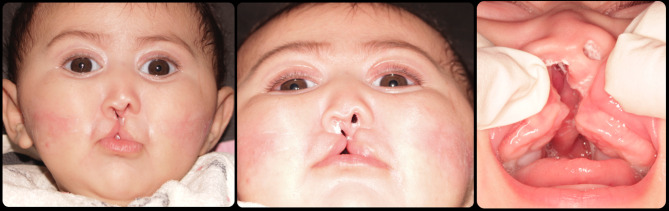
Case 1 with Tessier classification number 0

The first case is S.M., she is an Egyptian 2-week-old infant. She is suffering from a median cleft with congenitally missing premaxilla representing Tessier classification number 0 (Fig. 1). Her midface is severely retruded with collapsed zygoma. The 2 nostrils are deformed, and the columella is very thin, short and attached to the left labial segment (Fig. 1). Complete cleft palate is apparent too.

### Case 2

**Fig. 2:**
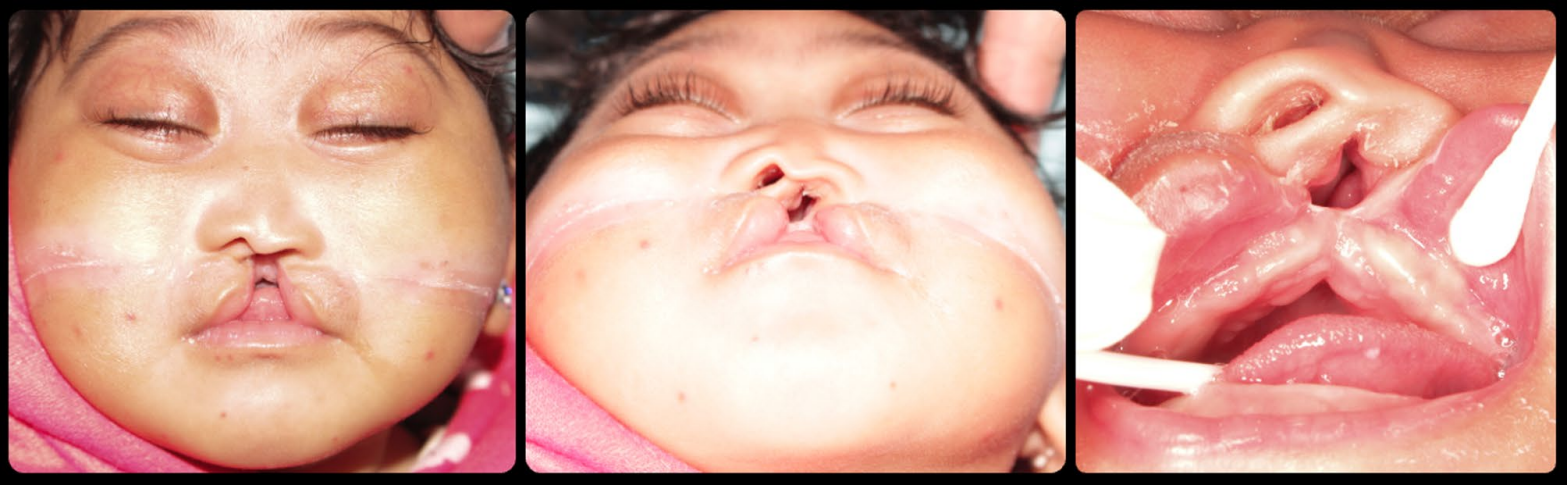
Case 2 with Tessier classification number 0

The second case is like case 1. F.A. came to the clinic at 1 week of age. She is also with a median cleft and congenitally missing premaxilla, midface deficiency and diminished zygoma representing Tessier classification number 0. Again, her nostrils are malformed with short and thin columella attached to the right labial segment and completely clefted palate (Fig. 2).

### Case 3

**Fig. 3:**
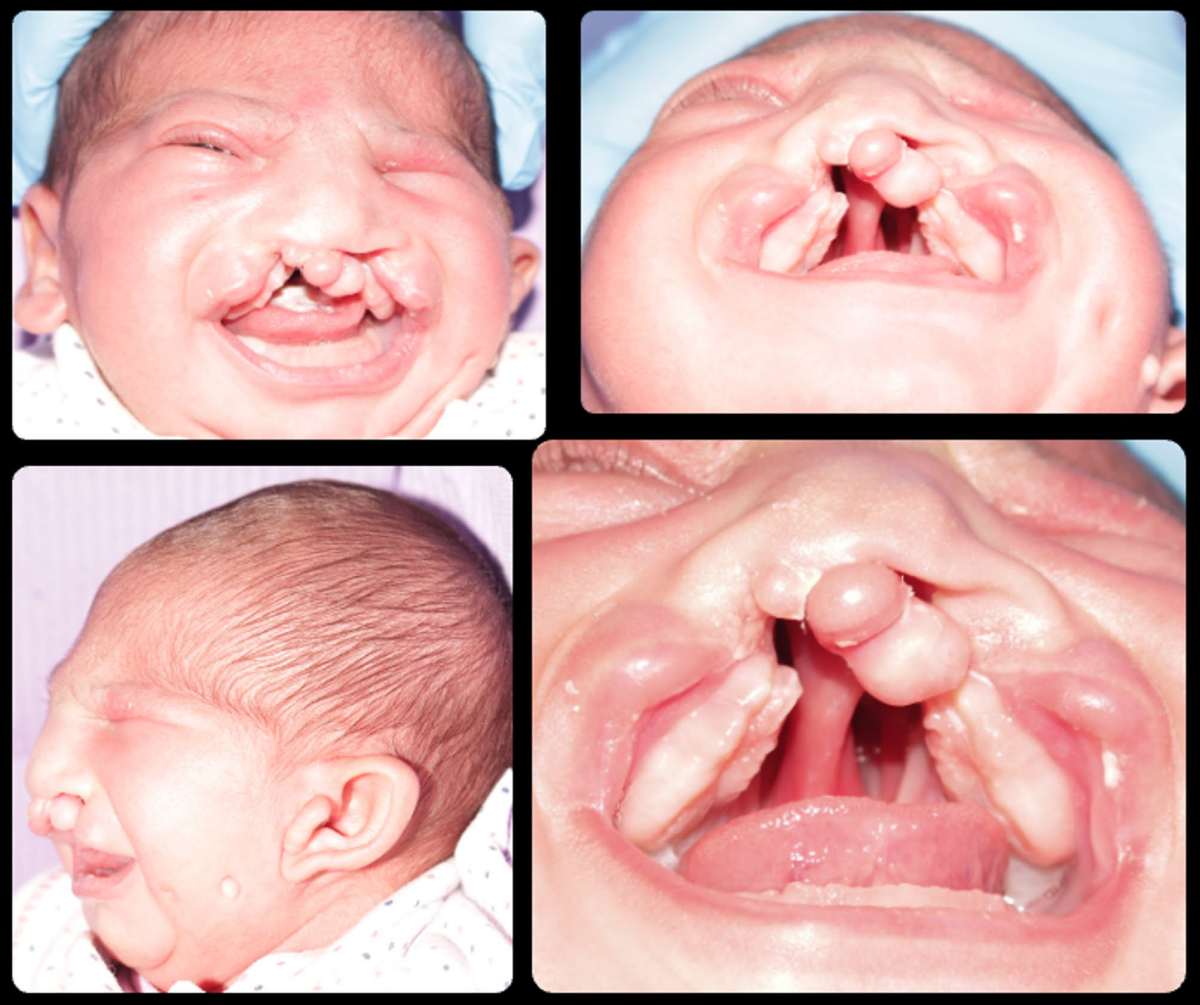
Case 3 with Tessier classification number 2 & 8

The third case is different, Z.A. was 10 days-old when she visited the clinic. She is with bilateral complete cleft lip and palate. The premaxilla is rotated to the left side with short prolabium. Her left ramus is obviously shorter than the right one and hemifacial microsomia with skin tags on the left cheek representing Tessier classification number 2 & 8 (Fig. 3).

### Case 4

**Fig. 4:**
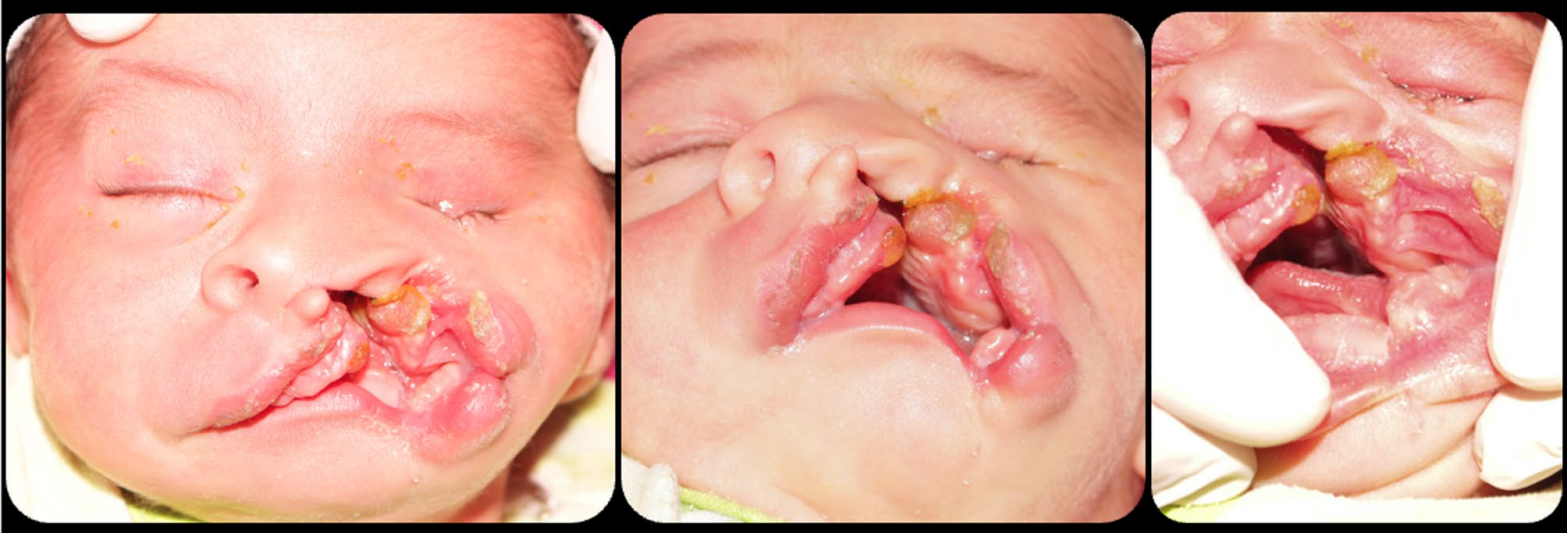
Case 4 with Tessier classification number 2

The next case is R.W. who presented at 12 days of age. She is suffering from unilateral complete cleft lip and palate in the maxilla. What makes this case very different is, the cleft is extended downward producing cleft in the lower lip as well as mandibular osseous cleft representing Tessier classification number 2 (Fig. 4). Her clefted nostril is severely deformed and fer left eyeball is underdeveloped.

### Case 5

**Fig. 5:**
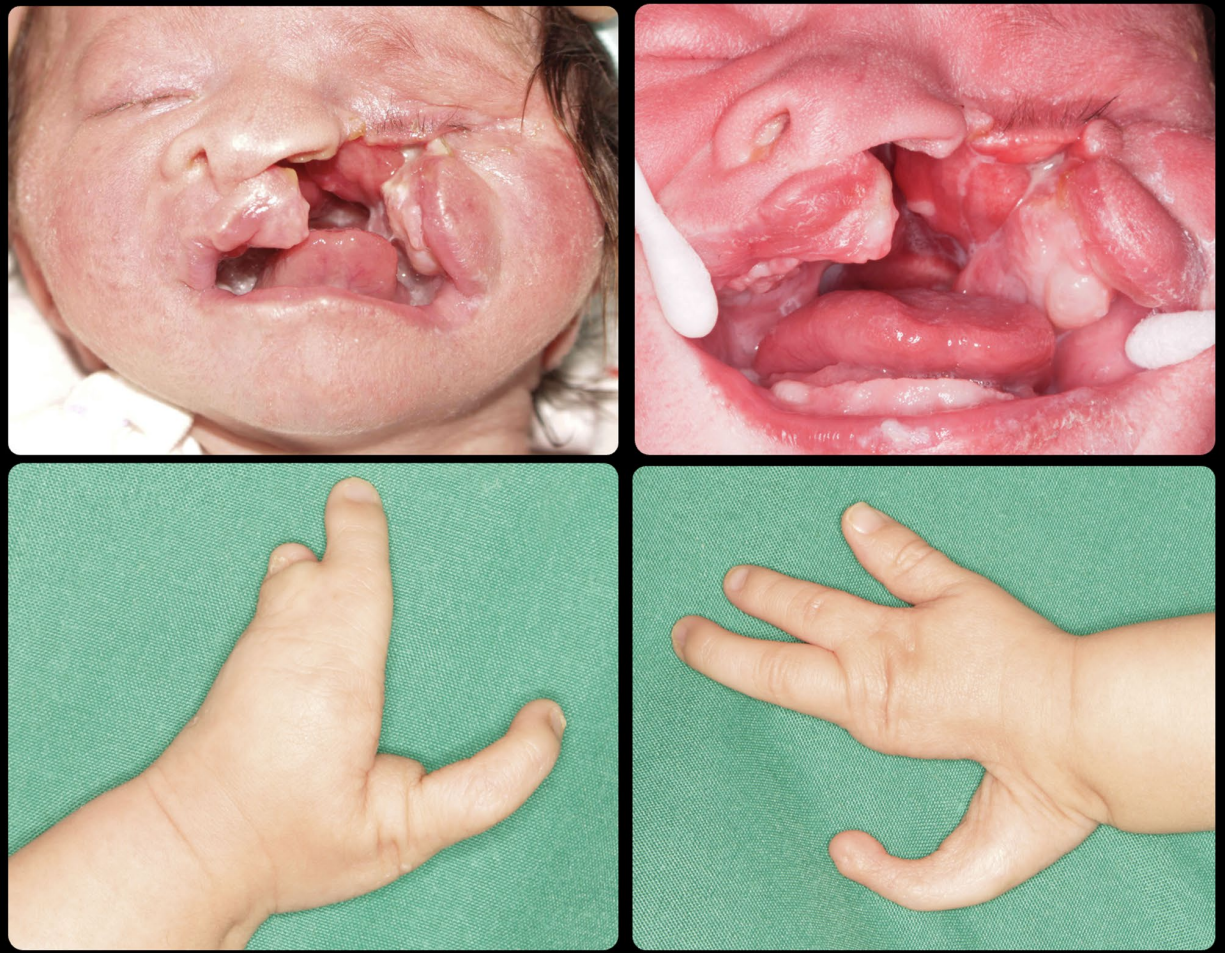
Case 5 with Tessier classification number 3

**Fig. 6:**
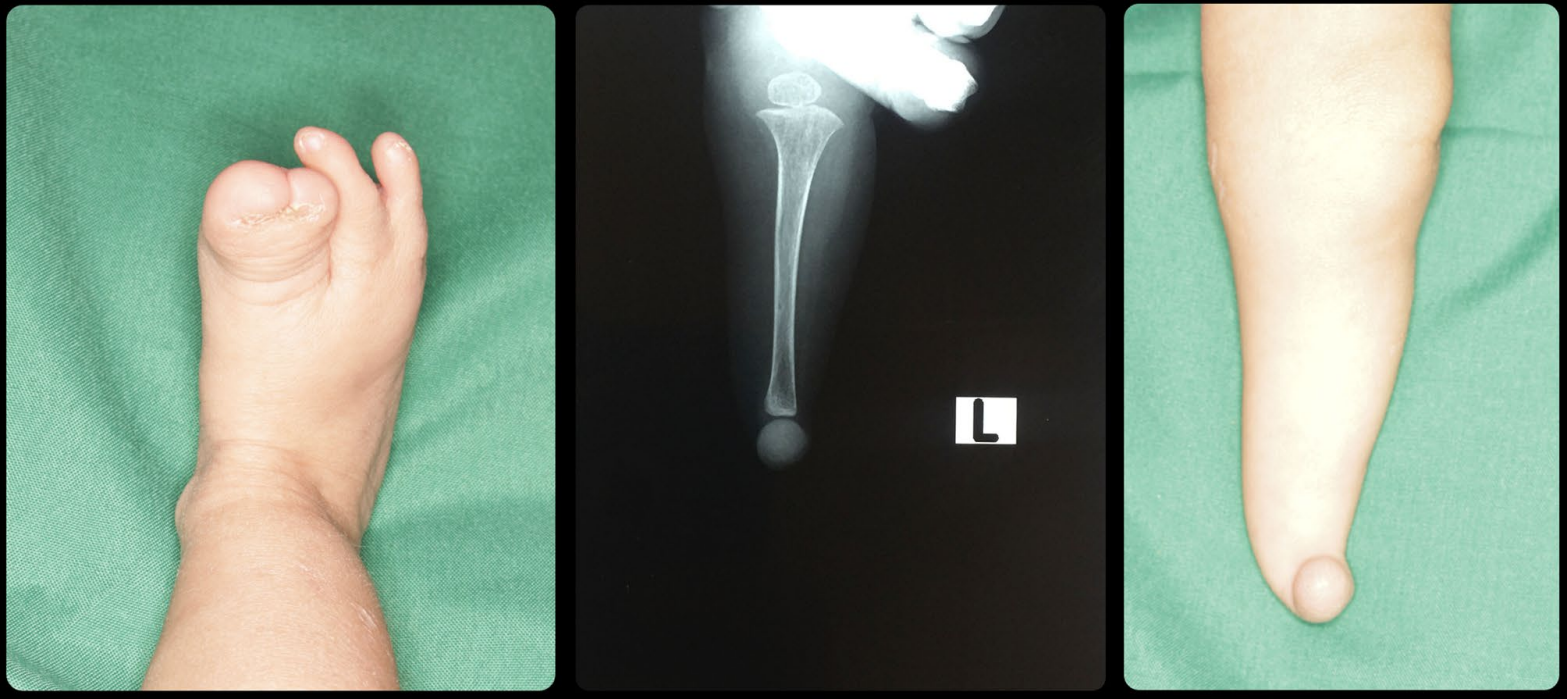
Cont. case 5

The last case and the most severe one is M.H. She visited the clinic at the age of 7 days. She is suffering from oblique facial cleft on the left side including the upper lip and extending upward resulting in complete loss of the left eyeball representing Tessier classification number 3 (Figs. 5 and 6). Besides the orofacial defect and the complete cleft palate, the 4 limbs are affected too. Severe deformities are present in the 2 hands in the form of fused and missing fingers. In lower limbs fusion and deformity of the toes of the right leg, in addition to complete absence of the left sole of the foot (Figs. 5 and 6).

### Detection of HCMV IgG

After patients’ presentation to the orthodontic clinic, all the mothers were referred for detection of HCMV IgG in blood samples. The drawn blood was transferred to vials to be ready for viral titer estimation using antibody testing. Using the centrifuging machine, serum was separated from the collected blood samples. HCMV IgG was detected in the serum samples using µ-capture enzyme immunoassay method (ELISA kits). Clinical trial number: not applicable.

## Results

**Fig. 7:**
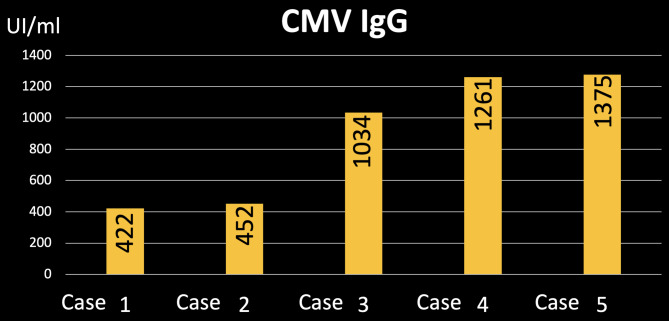
Amount of HCMV IgG in each case

HCMV IgG was positive for all the included mothers. The amount of HCMV IgG was the least in the least severe case (case 1) with the value of 422 UI/ml followed by case 2, 3 and 4 with the values of 452, 1034 and 1261, respectively. The highest value of the HCMV IgG was found in the most severe case (case 5) recording 1375 UI/ml (Fig. 7; Table 1). The HCMV IgG mean is 908.8 (± 400.6).

## Discussion

Identification of the possible etiological factors behind the incidence of cleft lip and palate is very beneficial. Knowing the etiological factors before birth might play a role in prevention of defect development or at least will provide more time to be prepared with the optimum interventions. Moreover, prediction of the defect severity is considered as another advanced diagnostic level that will be with great advantage if reached.

HCMV infection to the mothers before pregnancy can be considered as a hidden cause that can be easily present without any symptoms or clinical features. This article was aiming to report the association of HCMV infection to the mothers and the incidence of severe orofacial clefts.

In the current study, the 5 included patients were suffering from relatively severe orofacial clefts and all the patients’ mothers were with positive HCMV IgG. This result is matching what was found in the 3 previously published studies 14, 22, 23 in which it was found that there is a possibility association between HCMV infection to mothers before pregnancy and occurrence of cleft lip and palate.

The interesting result that was reached in the current study is the values of the serum HCMV IgG were higher in cases with more severe clinical presentation. This might prove more the association between HCMV infection and incidence of orofacial cleft and additionally, the association between the amount of the infection and the severity of the produced defects.

The current study is with some limitations, including the small sample size and the study design, both of which are due to the rarity of severe cases with orofacial cleft. Another possible limitations are the absence of genetic data and a control group for comparison. Moreover, the absence of data about HCMV IgG in the included infants and the timing of maternal HCMV infection are considered as study limitations. Because of these limitations, the generalizability of the results of the current study might be affected.

The results are highlighting the importance of detection of HCMV in the female’s serum before pregnancy and the impact of this dormant infection on the development of orofacial clefts.

## Conclusion

With the limitation of the data extracted from the 5 included cases the following can be concluded:

1. HCMV infection to mothers before or during pregnancy might be associated with the incidence of orofacial clefts.
2. Severity of orofacial clefts might be associated with severity of HCMV infection before or during pregnancy.
3. Detection of HCMV IgG in the mothers’ serum before pregnancy might be used as a predictor for incidence of cleft lip and palate to the future infants.

## Data Availability

The data underlying this article are available in the article and in its online supplementary material.
